# Control of gastrointestinal helminths in small ruminants to prevent anthelmintic resistance: the Italian experience

**DOI:** 10.1017/S0031182023000343

**Published:** 2023-10

**Authors:** Anna Maurizio, Stefania Perrucci, Claudia Tamponi, Antonio Scala, Rudi Cassini, Laura Rinaldi, Antonio Bosco

**Affiliations:** 1 Department of Animal Medicine, Production and Health, University of Padova, Viale dell'Università, 16-35020 Legnaro, Italy; 2 Department of Veterinary Sciences, University of Pisa, Viale delle Piagge 2, 56124 Pisa, Italy; 3 Department of Veterinary Medicine, Veterinary Teaching Hospital, University of Sassari, Sassari, Italy; 4 Department of Veterinary Medicine and Animal Production, CREMOPAR, University of Naples Federico II, 80137 Naples, Italy

**Keywords:** Anthelmintic resistance, Gastrointestinal helminths, small ruminants, sustainable parasite control, targeted (selective) treatments

## Abstract

Helminth infections are ubiquitous in grazing ruminants and cause significant costs due to production losses. Moreover, anthelmintic resistance (AR) in parasites is now widespread throughout Europe and poses a major threat to the sustainability of modern ruminant livestock farming. Epidemiological data on the prevalence and distribution of gastrointestinal nematodes, cestodes and liver- and rumen-flukes in Italian small ruminants are outdated and fragmentary. However, anthelmintics are commonly used to control these infections and often without prior diagnosis. Compared to other European countries, few reports of AR in small ruminants against levamisole, ivermectin and benzimidazoles have been published in Italy, but recent studies suggest that this phenomenon is spreading. Increased and integrated research efforts in developing innovative approaches to control helminth infections and AR are needed and must be tailored to the peculiarities of each context in order to be effectively implemented. This manuscript provides an overview on helminth prevalence and distribution, sustainable treatment strategies and integrated control approaches in small ruminants in Italy. The implementation of targeted treatments and targeted selective treatments is discussed based on different parameters, such as fecal egg count, degree of anaemia (FAMACHA^©^ method), milk production and body condition score. In addition, several Italian studies have also investigated the implementation of alternative strategies such as the use of different natural bioactive compounds or genetic selection for resistance and resilience to helminth infections. These concrete solutions for helminth management in small ruminant farms in the country are reported and discussed, representing a valid example for other Mediterranean countries.

## Graphical abstract

**Figure:**
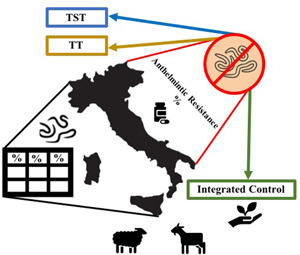

## Introduction

The small ruminant sector occupies a niche in the Italian livestock industry, accounting for about 1.3% of the total agricultural production. However, globally, Italian small ruminant farms stand out for their commercial and technical development, as well as for the quality of their products (e.g. sheep cheese with Protected Designation of Origin, Pecorino Romano) (Pulina *et al*., 2018). Moreover, this sector has historically played an important social and environmental role, especially in the alpine environments and in regions where production is more concentrated. In Italy, there are about 6.1 million sheep in 83 000 farms and 1 million goats in 52 000 farms (National Data Bank – NDB on 30th June 2022), mostly concentrated in the central and southern regions where the economical relevance of the sector is more pronounced.

Among the factors that threaten the sustainability of this sector, gastrointestinal helminth infections are known to be one of the major constraints due to their ubiquity, especially in grazing systems (Charlier *et al*., 2020). In general, their impact is primarily subclinical and associated with health and productivity losses and increased veterinary intervention costs. Because of their subclinical nature, the importance of these infections is often underestimated and overshadowed by other more evident health problems, which attract the attention of farmers. It is however estimated that in Italy alone, where the total annual revenue of the small ruminant sector amounts to 739 million € (Istituto di Servizi per il Mercato Agricolo Alimentare - ISMEA, 2022), the annual cost of helminth infections adds up to about 30 million € (attributable approximately half each to production losses and treatment costs), divided between dairy sheep (~22.5 million €), meat sheep (>6 million €) and dairy goats (>2.5 million €) (Charlier *et al*., 2020). The digestive system of small ruminants can be parasitized by a number of helminths which can have very different implications on the host in terms of type and intensity of pathogenic effect and on the control practices required. Knowledge about the presence and distribution of helminth infections is therefore crucial to plan effective parasite control programmes. However, epidemiological data on the distribution of gastrointestinal nematodes (GINs), cestodes and liver- and rumen-flukes in Italian small ruminants are outdated and fragmentary, as they are in other Mediterranean countries (Chartier and Reche, 1992; Valcárcel and García Romero, 1999; Papadopoulos *et al*., 2003; Kantzoura *et al*., 2012). Data on prevalence and intensity in Italy, published in scientific journals and conference proceedings over the last 10 years (2012–2022) for each helminth group are reported in Tables 1 and 2 for sheep and goats, respectively. These tables summarize the results of 27 studies in which a total of 44 807 sheep (from 3431 farms) and 8129 goats (from 409 farms) were sampled, and provide a good overview on the epidemiology of gastrointestinal helminths in a Mediterranean country. Overall, GINs were the most common helminth group, with individual prevalence rates of 61.5–89.4% in sheep and 47.8–100% in goats and even higher farm prevalence (85.4–100% in sheep and 83.7–100% in goats). GINs were also the most abundant group, with generally higher values of mean eggs per gram (EPG) of feces for goats (476–1291 EPG for individual mean data) compared to sheep (253–974 EPG for individual mean data). Other nematodes consistently showed very low mean output levels (<25 EPG in sheep and <84 EPG in goats) but a wide range of prevalence values. Only *Skrjabinema* spp. showed a very low prevalence in sheep (<3.3% farm prevalence). *Marshallagia marshalli* and *Capillaria* spp. were not reported in either ruminant species. *Moniezia* spp. was evaluated only qualitatively with 25–73% and 24–43% farm prevalence and 7–34% and 0–50% individual prevalence in sheep and goats, respectively. In particular, higher prevalence rates were reported for goats in southern Italy. Trematoda in goats occurred sporadically in the northern and central regions, whereas *Dicrocoelium dentriticum* and *Calicophoron daubneyi* were more common in the south. In sheep, the distribution of trematoda was homogeneous throughout the country, but even in this species *Fasciola hepatica* was rarely reported.

**Table 1.** Epidemiological studies on gastrointestinal helminths in sheep published in Italy over the last decade (2012–2022)

Geographic area (Italy)	Parasite																					References
	GINs		*Strongyloides papillosus*		*Nematodirus* spp.		*Skrjabinema* spp.		*Trichuris* spp.		*Moniezia* spp.		*Fasciola hepatica*		*Dicrocoelium dendriticum*		*Calicophoron daubneyi*		Sample size			
	P(%)	mean EPG	P(%)	mean EPG	P(%)	mean EPG	P(%)	mean EPG	P(%)	mean EPG	P(%)	mean EPG	P(%)	mean EPG	P(%)	mean EPG	P(%)	mean EPG	No. of animals	No. pooled samples	No. farms	
North	89.4	253.0	8.4	6.5	41.5	24.5	0.0	0.0	41.4	25.5	33.5	/	0.0	0.0	47.7	49.0	0.0	0.0	360	/	18	Midulla *et al*. (2014)
	99.0	/	83.0	/	73.0	/	0.0	/	52.0	/	38.0	/	6.0	/	24.0	/	0.0	/	1020	102	34	Zanzani *et al*. (2015)
	81.5	/	94.5	/	8.5	/	0.0	/	6.9	/	8.0	/	/	/	/	/	/	/	1698	68	68	Lambertz *et al*. (2018)
	/	/	/	/	/	/	/	/	/	/	/	/	0.0	/	2.7	/	0.0	/	1698	68	68	
Centre	94.0	152.4	0.0	0.0	35.8	3.0	0.0	0.0	37.3	4.2	65.6	/	1.5	0.3	26.8	7.6	4.5	1.2	1005	201	67	Culmone *et al*. (2012)
	/	/	/	/	/	/	/	/	/	/	/	/	/	/	22.0	/	/	/	5715	1143	381	Sanna et al. (2014)
	/	/	/	/	/	/	/	/	/	/	/	/	/	/	/	61.4	/	/	1815	363	121	
	85.2	547.7	18.1	/	0.0	/	0.0	/	19.7	/	6.5	/	0.0	/	32.8	/	0.0	/	61	/	/	Secchioni *et al*. (2016)
	91.4	342.4	0.0	/	33.7	8.6	0.0	/	35.3	15.2	57.1	/	0.7	/	24.6	12.7	7.0	/	/	431	320	Scala *et al*. (2018)
	66.7	334.8	4.8	3.9	0.0	0.0	0.0	0.0	5.0	2.9	10.5	/	0.0	/	0.0	/	0.0	/	600	/	12	Paoletti *et al*. (2018)
	/	/	/	/	/	/	/	/	/	/	/	/	/	/	/	/	51.1	31.2	1935	/	129	Scala *et al*. (2019)
South	91.8	/	4.1	/	24.5	/	2	/	39.8	/	35.7	/	1	/	61.2	/	10.2	/	1960	/	98	Bosco *et al*. (2013)
	61.5	/	/	/	/	/	/	/	/	/	/	/	/	/	/	/	/	/	314	/	/	Dipineto *et al*. (2013)
	90.5	219.9	/	/	/	/	/	/	/	/	/	/	/	/	/	/	/	/	/	/	21	
	/	428.7	/	/	/	/	/	/	/	/	/	/	/	/	/	/	/	/	984	/	54	Rinaldi *et al*. (2014)
	95.5	529.2	/	/	/	/	/	/	/	/	/	/	/	/	/	/	/	/	/	/	89	Rinaldi *et al*. (2015)
	100	414.0	40.0	2	73.3	4.0	0.0	0.0	46.7	4.0	73.3	/	0.0	0.0	0.0	0.0	0.0	0.0	300	/	15	Russo *et al*. (2016*a*)
	85.4	/	1.1	/	15.3	/	0.1	/	18.0	/	25.4	/	0.8	/	36.8	/	6.9	/	25 640	5128	1282	Bosco *et al*. (2017)
	100	378.0	56.7	6	50.0	4.0	3.3	1	23.3	2.0	70.0	/	3.3	11	30.0	2.0	13.3	1	600	/	30	Castagna *et al*. (2018)
	/	973.6	/	/	/	/	/	/	/	/	/	/	/	/	/	/	/	/	800	/	10	Bosco *et al*. (2020)
	/	/	/	/	/	/	/	/	/	/	/	/	/	/	/	/	7.9	76.0	/	/	682	Bosco *et al*. (2021)
tot																			44 807	7368	3431	

P(%), prevalence; mean EPG, mean eggs per gram of feces; GINs, gastrointestinal nematodes (*Haemonchus, Trichostrongylus, Teladorsagia, Cooperia, Oesophagostomum, Chabertia* and *Bunostomum*); /, data not available.

**Table 2.** Epidemiological studies on gastrointestinal helminths in goats published in Italy over the last decade (2012–2022)

Geographic area (Italy)	Parasite																					References
	GINs		*Strongyloides papillosus*		*Nematodirus* spp.		*Skrjabinema* spp.		*Trichuris* spp.		*Moniezia* spp.		*Fasciola hepatica*		*Dicrocoelium dendriticum*		*Calicophoron daubneyi*		Sample size			
	P(%)	mean EPG	P(%)	mean EPG	P(%)	mean EPG	P(%)	mean EPG	P(%)	mean EPG	P(%)	mean EPG	P(%)	mean EPG	P(%)	mean EPG	P(%)	mean EPG	No. animals	No. pooled samples	No. farms	
North	47.8	1291.1	15.8	68.4	0.3	33.4	25.4	/	7.1	84.0	10.0	/	/	/	/	/	/	/	379	/	13	Alberti *et al*. (2012)
	78.0	/	0.6	/	12.0	/	0	/	1.7	/	7.2	/	/	/	/	/	/	/	1838	55	55	Lambertz *et al*. (2018)
	/	/	/	/	/	/	/	/	/	/	/	/	0.0	/	0.2	/	0.0	/	1838	55	55	
	63.6	627.0	34.9	60.4	14.3	1.7	52.2	/	37.3	20.2	14.3	/	0.0	/	1.5	0.1	0.0	/	1675	335	53	Zanzani *et al*. (2018)
	37.9	484.2	28.4	25.3	0.4	0.1	18.9	12.5	8.0	7.0	0.0	0.0	/	/	/	/	/	/	264	/	13	Maurizio *et al*. (2021*a*)
Centre	100	/	/	/	/	/	/	/	/	/	/	/	/	/	/	/	/	/	320	/	31	Corrias *et al*. (2012)
	85.4	475.7	9.8	/	0.0	/	0.0	/	24.4	/	4.9	/	1	/	12.2	/	0.0	/	41	/		Secchioni *et al*. (2016)
South	100	683.0	40.2	2.0	70.0	4.0	0.0	0.0	60.3	10.0	50.2	/	0.0	0.0	0.0	0.0	40.3	15.0	160	/	10	Russo *et al*. (2016*b*)
	100	558.2	0.0	0.0	26.2	9.9	0.0	0.0	41.4	46.4	30.3	/	0.0	0.0	47.4	19.1	65.6	237.5	200	/	4	Castagna *et al*. (2014)
	100	633.0	0.0	0.0	26.6	18.0	0.0	0.0	42.2	16.0	29.2	/	0.0	0.0	46.9	8.0	65.1	124.0	192	/	4	Castagna *et al*. (2017)
	83.7	/	0.0	/	5.3	/	8.1	/	30.9	/	23.6	/	0.0	/	16.3	/	2.4	/	2460	492	123	Bosco *et al*. (2017)
	100	648.0	63.3	11.0	63.3	7	16.8	1	66.7	11.0	43.3	/	3.3	14.0	30.0	3.0	13.3	2.0	600	/	30	Castagna *et al*. (2018)
	/	/	/	/	/	/	/	/	/	/	/	/	/	/	/	/	2.7	45.0	/	/	73	Bosco *et al*. (2021)
tot																			8129	882	409	

P(%), prevalence; mean EPG, mean eggs per gram of feces; GINs, gastrointestinal nematodes (*Haemonchus, Trichostrongylus, Teladorsagia, Cooperia, Oesophagostomum, Chabertia* and *Bunostomum*); /, data not available.

For several decades, the control of these parasitic infections has mainly relied on the repeated use of chemical anthelmintics provided by pharmaceutical companies. A major issue for GIN control in small ruminants is treatment failure due to anthelmintic resistance (AR). Indiscriminate and/or inappropriate use of anthelmintic drugs to control GIN infections has led to selection of drug-resistant populations (Charlier *et al*., 2022). However, the accelerated development of AR in GIN populations and the extent of the phenomenon, including the increase of multi-resistant isolates, have been reported worldwide (Rose Vineer *et al*., 2020). Recent meta-analysis studies showed that AR is widespread in GINs in Europe, being reported in 5 economically important GIN genera (Haemonchus, Teladorsagia, Cooperia, Nematodirus and Trichostrongylus) and across 16 European countries (Rose Vineer *et al*., 2020). Results revealed an average farm level prevalence of AR to benzimidazoles (BZ) of 48 and 51%, to macrocyclic lactones other than moxidectin (MLs) of 29 and 44%, and to levamisole of 32 and 20%, in sheep and goats, respectively (Rose Vineer *et al*., 2020; Charlier *et al*., 2022).

In Italy, few reports of AR in small ruminants against levamisole, macrocyclic lactones and benzimidazoles have been published (Table 3). The first reports of AR in small ruminants date back to 2007 for benzimidazoles, levamisole and ivermectin in central and southern Italy (Cringoli *et al*., 2007; Traversa *et al*., 2007). Subsequently, 3 studies conducted in northern Italy in sheep and goats reported additional cases of AR for benzimidazoles, levamisole, ivermectin, moxidectin and eprinomectin (Zanzani *et al*., 2014; Geurden *et al*., 2015; Lambertz *et al*., 2019). The last report of AR was recorded in southern Italy in some sheep farms for albendazole (Bosco *et al*., 2020), although the phenomenon had not occurred in previous years in the same area (Rinaldi *et al*., 2014). This means that AR will inevitably spread. For this reason, increased and integrated research efforts are needed in the development of innovative approaches to control helminth infections and AR that must be tailored to the peculiarities of each context in order to be effectively implemented.

**Table 3.** Reports of anthelmintic resistance in small ruminants in Italy

Animal species	Anthelmintics or anthelmintic class	Geographic area	Reference
Goat	Netobimin	Southern Italy	Cringoli *et al*. (2007)
Sheep	Benzimidazoles Levamisole Ivermectin	Central Italy	Traversa *et al*. (2007)
Goat	Benzimidazoles Eprinomectin	Northern Italy	Zanzani *et al*. (2014)
Sheep	Benzimidazoles Levamisole	Northern Italy	Geurden *et al*. (2015)
Goat	Benzimidazoles Levamisole Ivermectin Moxidectin Eprinomectin	Northern Italy	Lambertz *et al*. (2019)
Sheep	Albendazole	Southern Italy	Bosco *et al*. (2020)

This article provides an overview on targeted (selective) treatments (TT/TST), sustainable and integrated control approaches in small ruminants in Italy. The Italian perspective could be a concrete example for other Mediterranean countries, with similar climatic conditions and farm management, to analyse and control gastrointestinal helminth infections and the possible presence of AR in their realities.

## Targeted selected treatment

The targeted selective treatments (TST) were introduced with the aim of reducing the number of animals treated within a flock. In this approach, only those animals that benefit most from treatment or contribute most to pasture contamination (usually a small percentage of the flock) are treated, leaving the rest of the flock untreated (van Wyk and Bath, 2002).

The concept behind this strategy is to ensure the maintenance of parasite that are not exposed to the selective pressure of anthelmintics (i.e. ‘*refugia*’), both as larval stages in pastures and as immature/adults in untreated animals (Kenyon *et al*., 2009; Medina-Pérez *et al*., 2015).

Nowadays there is a consensus among parasitologists in considering the parasites *in refugia* as a key factor in AR prevention because this population fraction will maintain the genes for susceptibility within the parasite population, thus competes with and limits the spread of resistant parasitic strains in treated animals (van Wyk, 2001; Kenyon *et al*., 2009).

Several markers can be used as indicators for TST. They are broadly classified into pathophysiological markers (such as anaemia, body condition and dag score), parasite-based markers (such as fecal egg count, FEC), and production parameters (such as milk production, wool production and live weight gain) (van Wyk and Bath, 2002; Kenyon *et al*., 2009; Riley and van Wyk, 2009; Charlier *et al*., 2014).

An ideal indicator for use in TST would be cost-effective, simple to use, require minimal operator training and allow treatment decisions to be made ‘sheep-side’ (Kenyon *et al*., 2009). It must be chosen according to the parasitological situation present (prevalent nematode species), type of production (meat, milk or wool), available resources of the farm (Charlier *et al*., 2014; Chylinski *et al*., 2015).

Over the years, several studies have addressed this issue to identify the best indicators. The degree of anaemia caused by the bloodsucking nematode, *Haemonchus contortus* is used as a TST indicator in the FAMACHA^©^ method. In this method, the colour of the conjunctival mucosal membrane is assessed through a 5-point scale. Animals with score 4 or 5 on the scale are considered at risk of disease and should be treated (Bath *et al*., 2001; Vatta *et al*., 2001). The FAMACHA^©^ method has been used successfully in many countries (Vatta *et al*., 2001; Burke *et al*., 2007; Mahieu *et al*., 2007) and has been proven to be beneficial, especially for resource-poor farmers, but presents some limitations related to the ubiquity of mixed nematode infections and the presence of other blood-feeding parasites, e.g. liver flukes (Papadopoulos *et al*., 2013; Charlier *et al*., 2014). The first study carried out in Italy to evaluate the usefulness of the FAMACHA^©^ method for detecting the severity of anaemia in sheep highlighted a low sensitivity of this system in detecting anaemic sheep, based on the comparison with a complete haematological profile obtained from sheep with GIN infection (Di Loria *et al*., 2009). The results of the study suggested that, in the specific context of sheep farming in southern Italy, the FAMACHA^©^ method may represent an additional element to integrate clinical examinations but cannot be used as a parameter for TST. Local problems may have affected the accuracy of this method in the study area, e.g. the quantitative parasitological status of the animals, the pathogenesis and virulence of the parasite strains, the local epidemiology, the simultaneous presence of other bloodsucking parasites like liver fluke, the treatments, the breed and the physiological status of animals, feeding, etc. The Authors also suggested that further long-term studies should be conducted in other areas of Italy to determine the validity of this approach as TST indicator.

FEC, employed with different threshold values, is an effective parameter but presents several shortcomings, such as the need for individual examination of fecal samples that is expensive and time-consuming, and must be performed in the laboratory, making the treatment decision not immediate or ‘sheep-side’ (Charlier *et al*., 2014). It is also challenging when applied in large flocks because egg excretion depends on the physiological situation of individual animals (e.g. after lambing, related to the post-parturient rise) and the nematode species present, e.g. *H. contortus* females are particularly fecund, resulting in high FECs even when worm burdens are low (Kenyon *et al*., 2009; Arece-García *et al*., 2016). Production indicators have been largely used for TST in sheep because they are often easier to measure on an individual basis and are widely preferred by farmers, allowing equipment and labour to be justified by broader use than just parasite control (Charlier *et al*., 2014). Milk production has been successfully applied, especially in goats, with limiting treatment to high producers and animals in their first lactation resulting in similar levels of egg excretion as mid-season systematic treatment of all animals (van Wyk *et al*., 2006; Charlier *et al*., 2014). Weight gain can also be used as an effective indicator for TST and is appreciated for automation in situations where the cost of time and labour required for individual animal inspection is prohibitive (Stafford *et al*., 2009). A study conducted in southern Italy involving 4 sheep farms compared TST with strategic treatment. FAMACHA^©^, FEC and milk production were used as TST indicators. The findings from this study showed that by adopting a TST approach it was possible to reduce drug usage by 40–60%, suggesting the economic profitability of this approach also in the Italian context, even though the costs for the additional diagnostic efforts were not considered in the analysis. Among the different TST indicators used, milk production might be the most appropriate one, given the importance of sheep milk production in southern Italy (Cringoli *et al*., 2009).

Finally, body condition score (BCS) is considered one of the simplest and most cost-effective indicators of an animal's fat reserve that can be used during periods of high-energy demand, stress and/or sub-optimal nutrition as is for example characteristic of GIN infections (van Wyk *et al*., 2006). BCS can be used as a TST indicator especially in infections with *Trichostrongylus* spp., *Teladorsagia* spp. and other non-bloodsucking nematodes known to reduce body weight (Bath *et al*., 2001), but has been seen to have some limitations, such as the need to adapt the treatment threshold to the physiological (or productive) situation in a farm (Soto-Barrientos *et al*., 2018; Aguirre-Serrano *et al*., 2020). A study carried out in Sardinia Island (Tamponi *et al*., 2022) aimed to evaluate the use of BCS as a marker of TST in lactating sheep in 2 different lactation periods (third and fifth month of lactation) by comparing BCS with FEC. The results of the study showed a negative correlation (*r* = − 0.163) between EPG values and BCS of the studied animals, with the highest EPG values occurring in animals with the lowest BCS. The study confirmed that the use of TST in lactating ewes with a BCS <2.25, especially in older ewes, could be beneficial for subclinical GIN infections (*Teladorsagia* spp. and *Trichostrongylus* spp. were the most commonly identified GIN genera). However, further studies are needed to elaborate tailored recommendations for the broader system of small ruminants farming in the different regions of Italy.

## Targeted treatment

Besides TST, other drenching strategies such as whole-flock targeted treatments (TT) are available to control nematode infections. TT involves treating the whole flock based on knowledge of risk or parameters that quantify the severity of infection (Charlier *et al*., 2014). TT differs from whole-flock metaphylactic treatments, which are also used to protect animals and prevent disease over time, in its aim of reducing the number of treatments given to a flock and thus of minimizing the establishment of resistant genotypes in favour of susceptible worms (Kenyon *et al*., 2009). When performing TT, the concept of *refugia* remains, but unlike TST, the source of *refugia* is almost entirely limited to larval stages present in the environment at the time of treatment, which are the source of re-infection (Kenyon *et al*., 2013). The use of TT was proved to be effective in controlling GIN infections and slowing the onset of AR (Kenyon *et al*., 2013). In a study carried out in a dairy goat farm in southern Italy, using 5 different drenching protocols based on 2 treatments (i.e. the first with ivermectin, the second with netobimin) administered at different times of the year, all the treated groups showed a total milk production higher than the control group (Veneziano *et al*., 2004). The best timing of treatment in terms of increased milk production was October–May, followed by February–June, but the results indicated that the second treatment, performed either in May or in June, during the lactation period, seemed to be mandatory to maintain the performances. Of concern is that the only anthelmintic available in Italy without a withdrawal period for milk in small ruminants is currently eprinomectin, which imposes significant limitations on drug rotation. In the same region, the use of TT was tested in 4 commercial dairy sheep farms in relation to parturition. A 2-treatment protocol was used, the first in the periparturient period and the second in mid/end lactation (Cringoli *et al*., 2008). Drugs from 2 different anthelmintic classes were employed (i.e. moxidectin for the first drenching and netobimin for the second). This strategic approach improved milk yield of naturally infected ewes by 19–44%, compared to untreated animals, and proved to be economically effective. In particular, moxidectin appeared to assist the ability of the host to limit egg excretion in the periparturient period and to have a sort of prophylactic effect, which was also confirmed in a later study (Cringoli *et al*., 2009). TT can also be administered to growing lambs in conjunction with the weaning phase (Kenyon *et al*., 2013) or pasture management (Besier, 2012), but no specific studies have been conducted on this in Italy.

An alternative to avoid linking treatment to specific seasons of the year or production cycles, is the use of parasitological indicators of infection burden. FEC techniques (e.g. Mini-FLOTAC, McMaster techniques) remain the most common laboratory methods for the quantitative diagnosis of gastrointestinal helminths and may offer benefits as they can allow treatments to be adapted to seasonal and temporal changes in parasite burden (Charlier *et al*., 2014). When used for the purpose of monitoring the flock burden, samples are collected from a small number of animals (e.g. 10–20) and used to estimate the whole-flock burden. However, sample size affects the accuracy of estimation of group mean FEC. Reducing the sample size in order to contain the costs may lead to underestimation (Morgan *et al*., 2005) as a result of the aggregated distribution that parasites display in the host population. For this reason, a formula was developed and tested in 13 dairy goat farms (264 sampled animals) in northern Italy to tailor the sample size on the farm size, keeping an account of the expected mean, aggregation and the desired precision (Maurizio *et al*., 2021*a*). This formula allows the calculation of the minimum sample size to achieve a good accuracy in the burden estimation, while providing information on the parasite distribution within that flock. An alternative is the use of pooled samples, which however lacks to provide this last information but is very effective in reducing the costs of analysis (Rinaldi *et al*., 2019). As mentioned earlier, the FEC techniques are often criticized for poorly correlating with actual infection levels, health status, and animal performance. For this reason, and given the many factors that can influence FEC outcomes, strict thresholds for treatment should not be applied and monitoring should continue periodically to assess baseline FEC (Sargison, 2013; Maurizio *et al*., 2021*b*). The sample size is also critical for evaluating the anthelmintic efficacy as demonstrated by a recent study that reported a new approach for determining sample size requirements for the fecal egg count reduction test (FECRT) that is built on a solid statistical framework (Denwood *et al*., 2023).

## Alternative and complementary approaches

Although the use of chemical anthelmintics is still the most common approach used by Italian farmers to control helminths in livestock, alternative or complementary methods are also available, which include nutritional, immunological and biological interventions, as grazing management, nutritional supplementation, genetic selection, biological control and vaccination (Charlier *et al*., 2022). Among these approaches, the use of natural bioactive compounds is also considered a valid option due to their anti-parasitic properties (Hoste *et al*., 2015). Natural bioactive compounds, such as plant extracts and plant-derived compounds can be used as herbal medicines or nutritional supplementations, or as models for the synthesis of new drugs (Hoste *et al*., 2022).

In the following section, the studies conducted in Italy on the application of alternative and/or complementary methods to the use of anthelmintics for the control of helminth infections in ruminants are described and discussed. In particular, many plants are used in ethnoveterinary medicine for parasite control in small ruminants (Castagna *et al*., 2021) and strong and rooted ethnoveterinary knowledge is available in the country.

### Nutritional supplementation and phytotherapy

The results of the *in vitro* and *in vivo* tests evaluating the efficacy of natural extracts and pure compounds against GINs in small ruminants are briefly reported in Tables 4 and 5, respectively. These products were selected considering the content in secondary metabolites potentially active against GINs and the anthelmintic activity against other nematode species as reported in previous studies. Except for *Hypoestes forskaolii* extracts and some of the commercial pure compounds, all extracts and most pure compounds tested in these studies were obtained/derived from plants grown in Italy or present on the Italian territory.

**Table 4.** *In vitro* anthelmintic efficacy of plant-derived compounds against sheep gastrointestinal nematodes

Compounds	Origin of the principles	Dosage	Laboratory test	Target parasitic genera	Description of the outcomes	References
Saponin mixtures after extraction and purification	*Medicago* spp. plants	From 10 to 0.05 mg/mL	EHT	*Trichostrongylus*, *Oesophagostomum*, *Cooperia*, *Haemonchus* and *Chabertia ovina*	Inhibiting effects on GIN eggs in a concentration-dependent manner (according to the *Medicago* species 100% inhibition obtained at 2.5, 5 or 10 mg/ml)	Maestrini *et al*. (2020)
Aqueous extract containing 10% of glycyrrhizic acid	*Glycyrrhiza glabra* roots	30, 10, 5, 1, and 0.5 mg/mL	EHT, LDT, LMIT	Mainly *Trichostrongylus* and *Teladorsagia/ Ostertagia*	Highly effective at EHT, with a marked concentration-dependent effect; limited efficacy at LDT and LMIT	Maestrini *et al*. (2021)
Glycyrrhetinic acid	Pentacyclic triterpenic organic acid considered to be the major active component of *G. glabra* root aqueous Extract	30, 10, 5, 1, and 0.5 mg/mL	EHT, LDT, LMIT	Mainly *Trichostrongylus* and *Teladorsagia*/ *Ostertagia*	Highly effective at EHT, with a marked concentration-dependent effect; good efficacy at LDT and LMIT	Maestrini *et al*. (2021)
Hydroalcoholic extracts from basal and cauline leaves and flowers	*Isatis tinctoria*	1.00, 0.5, 0.25, 0.125 mg/mL	EHT	*Trichostrongylus, Haemonchus contortus, Teladorsagia, Chabertia ovina* and *Cooperia*	Highly effective	Ragusa *et al*. (2022)
N-hexane, chloroform, chloroform:methanol 9:1, and methanol crude extracts	*Hypoestes forskaolii*	1.00, 0.5, 0.25, 0.05 and 0.005 mg/mL	EHT	*Trichostrongylus, Haemonchus contortus, Teladorsagia, Chabertia ovina* and *Cooperia*	Only n-hexane extract showed a rather weak efficacy with a dose-dependent effect	D'Ambola *et al*. (2018)
Methanol extract and essential oil	*Ruta chalepensis*	0.02–0.20 and 0.40–6.30 mg/mL	LAPAMT	*Trichostrongylus*, *Haemonchus contortus*, *Teladorsagia*	Good anthelmintic efficacy	Ortu *et al*. (2017)
2-Undecanone, 2-nonanone, 2-decanone	*Ruta chalepensis*	0.03–1.60 (2-nonanone), 0.40–3.90 (2-undecanone) and 0.02–0.65 (2-decanone)	LAPAMT	*Trichostrongylus, Haemonchus contortus, Teladorsagia*	Good anthelmintic efficacy	Ortu *et al*. (2017)
Essential oils	*Origanum vulgare, Foeniculum vulgare, Satureja montana, Satureja hortensis* and 2 types of *Thymus vulgaris*	50, 12.5, 3.125, 0.781, 0.195 and 0.049 mg/mL	EHT	*Haemonchus, Trichostrongylus, Teladorsagia and Chabertia ovina*	All highly effective at the lowest concentration tested (0.049 mg/mL)	Štrbac *et al*. (2022*a*, 2022*b*)
Plant-derived pure compounds	Mangiferin, rutin, quercetin, and *β*-sitosterol	Mangiferin (0.25%, 0.125%, 0.0625%), rutin (1%, 0.75%, 0.5%), quercetin (1%), *β*-sitosterol (1%, 0.75%, 0.5%)	EHT, LDT, LPAMT	*Trichostrongylus, Haemonchus, Teladorsagia, Chabertia ovina*	Weak efficacy in EHT; good efficacy at LDT; rutin and mangiferin good efficacy at LPAMT	Giovanelli *et al*. (2018)
Plant-derived pure compounds	Aloin and aloe-emodin	Aloin (0.5%), aloe-emodin (0.1%)	EHT, LDT, LPAMT	*Trichostrongylus, Haemonchus, Teladorsagia*	Weak efficacy in EHT; good efficacy at LDT; good efficacy of aloin at LPAMT	Fichi *et al*. (2017)
Two sea buckthorn berry juices (SBT and SBF) commercialized for human consumption	*Hippophae rhamnoides*	2400, 1200, 600, 300 and 150 *μ*g/mL	EHT, LEIA	*Trichostrongylus, Oesophagostomum, Haemonchus*	Good efficacy of SBT at EHT; good efficacy of SBT and SBF at LEIA	Maestrini *et al*. (2022)
Methanolic fraction, insoluble residue, and gallic acid	*Punica granatum*	1.00, 0.5, 0.25, 0.125, 0.05 and 0.005 mg/mL	EHT	*Trichostrongylus, Haemonchus contortus, Teladorsagia, Chabertia ovina and Cooperia*	Highly effective	Castagna *et al*. (2020)

EHT, egg hatch test; LDT, larval development test; LMIT, larval migration inhibition test; LPAMT, larva paralysis and motility test; LEIA, larval-exsheathment inhibition assay.

**Table 5.** *In vivo* anthelmintic efficacy of plant-derived compounds in sheep naturally infected by gastrointestinal nematodes

Formulation	Origin of the formulation	Experimental settings	Type of test	Target parasitic genera	Efficacy mean FECR	References
Aqueous macerates	Bark and leaves of *Salix caprea*	15 Animals naturally infected with GINs treated with 50 mL of macerate as single dose by oral administration	FECRT at days 7, 14 and 21 after the treatment	GINs	0.1%	Castagna *et al*. (2021)
Aqueous macerates	Whole plant *Artemisia campestris*	16 Animals naturally infected with GINs treated with 50 mL of macerate as single dose by oral administration	FECRT at days 7, 14 and 21 after the treatment	GINs	20%	Castagna *et al*. (2021)
Aqueous macerates	Whole fruit (seeds and peel) of *Punica granatum*	17 Animals naturally infected with GINs treated with 50 mL of macerate as single dose by oral administration	FECRT at days 7, 14 and 21 after the treatment	GINs	50%	Castagna *et al*. (2021)
Aqueous macerate	Fruits and fruit rind of *P. granatum*	Two groups of 15 animals naturally infected with GINs treated with 50 mL of macerate as single dose by oral administration	FECRT at days 7, 14 and 21 after the treatment	*Haemonchus, Trichostrongylus, Teladorsagia* and *Chabertia*	54% (at days 14)	Castagna *et al*. (2022)
Essential oil	*Thymus vulgaris*	12 Animals naturally infected with GINs treated with 100 mg/kg of body weight	FECRT at days 7 and 14 after the treatment	*Haemonchus, Trichostrongylus, Teladorsagia* and *Chabertia*	39.30% (at days 7) <10% (at days 14)	Štrbac *et al*. (2022*a*, 2022*b*)
Combination of 2 pure compounds	Linalool + estragole	12 Animals naturally infected with GINs treated with 100 mg/kg of body weight	FECRT at days 7 and 14 after the treatment	H*aemonchus, Trichostrongylus, Teladorsagia* and *Chabertia*	51.9% (at days 7) <10% (at days 14)	Štrbac *et al*. (2022*a*, 2022*b*)

FECRT, fecal egg count reduction test; GINs, gastrointestinal nematodes.

In the study by Maestrini *et al*. (2020), saponin mixtures from *Medicago* spp. plants showed inhibiting effects on GIN eggs in a concentration-dependent manner and most of them showed an efficacy comparable to the reference drug (thiabendazole, TBZ) at higher concentrations. Biological effects of saponins are usually attributed to their specific interaction with cell membranes, causing changes in cell permeability (Cavalcanti *et al*., 2016). In a second study, the *in vitro* efficacy of an aqueous extract from *Glycyrrhiza glabra* roots (containing 10% glycyrrhizic acid) and of glycyrrhetinic acid on GINs of sheep was tested (Maestrini *et al*., 2021). Both products were highly active against eggs, but against larvae glycyrrhetinic acid showed a significantly higher anthelmintic efficacy than that of *G. glabra*, and its activity was comparable to that of thiabendazole at the highest concentration. Recently, a beneficial effect of dietary supplementation with *G. glabra* roots on the chemical and physical properties of cow's milk and cheese was reported (Bennato *et al*., 2019). Moreover, glycyrrhizic acid has been found able to modulate rumen bacterial flora with positive effects on the ruminal concentrations of total volatile fatty acids, acetate, propionate, and butyrate in sheep (Guo *et al*., 2019).

In another *in vitro* study (Ragusa *et al*., 2022), hydroalcoholic extracts of *Isatis tinctoria* leaves and flowers were found to be highly effective in inhibiting GIN egg hatching in sheep. The authors consider the possibility of using the hydroalcoholic extracts for treating infected sheep or the entire parts of *I. tinctoria* as a feed or dietary supplement in infected sheep for GIN control. In contrast, the study by D'Ambola *et al*. (2018) demonstrated weak ovicidal activity of *Hypoestes forskaolii* extracts against GIN eggs (2018).

The anthelmintic activity of 2 extracts and some pure constituents from *Ruta chalepensis L.* were also evaluated *in vitro* (Ortu *et al*., 2017) on GIN third stage larvae. Both extracts of *R. chalepensis* showed a significant anthelmintic activity against GINs *in vitro*, but they were less active than the conventional anthelmintic (levamisole). Moreover, their constituents 2-undecanone, 2-nonanone, 2-decanone, were found the most active *R. chalepensis* essential oil pure compounds.

The essential oils of *Origanum vulgare, Foeniculum vulgare, Satureja montana, Satureja hortensis* and 2 types of *Thymus vulgaris* were found to be very effective against GIN sheep eggs *in vitro* and, according to the authors (Štrbac *et al*., 2022*a*, 2022*b*), all of these essential oils could represent a promising research topic for further studies.

Among the pure active compounds, the *in vitro* anthelmintic activity of the plant-derived mangiferin, rutin, quercetin, *β*-sitosterol, aloin and aloe-emodin on sheep GIN eggs and larvae was investigated (Fichi *et al*., 2017; Giovanelli *et al*., 2018). On eggs, none of the tested compounds showed an efficacy comparable to that of the reference drug (thiabendazole, TBZ). With the exception of quercetin, all compounds completely prevented GIN larval development, showing an efficacy comparable to that of thiabendazole, while only rutin, mangiferin and aloin were effective as the reference drug in causing the death of third-stage larvae (L3) at the highest concentrations used.

The *in vitro* anthelmintic activity against sheep GIN eggs and larvae of 2 sea buckthorn berry juices (*Hippophae rhamnoides*) (SBT and SBF) commercialized for human consumption, was reported by Maestrini *et al*. (2022). At the highest concentration, the activity of SBT on eggs was high and comparable to that observed in the TBZ-treated controls, while SBF showed a lower efficacy. However, the 2 juices were found both effective on larvae. The polyphenols identified in higher concentrations in the 2 *H. rhamnoides* juices investigated in this study were glycosylated forms of isorhamnetin and, to a lesser extent, quercetin.

The *in vitro* activity of the methanolic fraction, insoluble residue, and gallic acid extracted from an aqueous *Punica granatum* macerate was also evaluated against sheep GIN eggs (Castagna *et al*., 2020). All fractions caused a significant GIN egg hatch inhibition (>82%) at all tested doses and comparable to that of TBZ, but the methanol fraction showed the highest efficacy, followed by the insoluble residue and gallic acid.

Despite evidence of anthelmintic properties, it is important to emphasize that results obtained *in vitro* are not directly applicable *in vivo* and compounds need to be tested for their safe and effective farm application. Several studies already investigated the *in vivo* use of some compounds in Italy.

The study by Castagna *et al*. (2021), showed significant *in vivo* anthelmintic activity against GINs of *Punica granatum* macerate, but low efficacy of *Artemisia campestris*, and a complete inefficacy of *Salix caprea*. The detected components of *P. granatum* macerate were mainly alkaloids, tannins, flavonoids, glycosides, and phenols, such as gallic and ellagic acid, whose reported anthelmintic activity could explain the anthelmintic efficacy of this extract (Dkhil, 2013; Ahmed *et al*., 2020). The *in vivo* anthelmintic efficacy of the pomegranate (*P. granatum*) macerate was confirmed in a further study (Castagna *et al*., 2022) that evaluated and compared the effects of a single oral dosage of 50 mL of this plant extract in 2 farms of sheep naturally infected with GINs. In both farms, the pomegranate macerate showed an efficacy of about 50% from day 7 to day 21 after the treatment. All these *in vivo* studies (Castagna *et al*., 2021, 2022) suggest that this aqueous pomegranate macerate could be as a sustainable alternative to the use of synthetic drugs to reduce the increase in AR and the environmental impact of anthelmintic drugs.

Štrbac *et al*. (2022*a*) evaluated the *in vivo* anthelmintic potential of a *T. vulgaris* essential oil and of the combination of 2 pure compounds (linalool and estragole) in 2 sheep farms with natural mixed GIN infections. The average percentage of individual EPG reduction in the groups treated with *T. vulgaris* and linalool:estragole reached 39.30% and 51.88% on day 7, respectively, but it was less than 10% on day 14.

Regarding dietary supplementation, the study by Castagna *et al*. (2019) evaluated the efficacy of a commercially available natural plant mixture, composed of essential oils and other extracts of plants belonging to the Compositae, Cesalpinaceae, Liliaceae, Bromeliaceae and Labiatae families, approved for the treatment of GIN infections in sheep at the doses recommended by the manufacturer. However, the results showed that the natural supplement was ineffective against GIN infections in sheep.

### Evaluation of selecting for resistance/resilience to helminth infections

Host resistance (i.e. the ability to mount an effective immune response) and resilience (i.e. the ability to produce even under worm challenge) to helminth infections are largely influenced by genetic factors, and can vary among species, breeds and individuals (Zvinorova *et al*., 2016; González *et al*., 2019). Resistance/resilience to helminths may increase productivity under conditions of high parasite infection risk and, because this leads to less frequent treatments, reduce selection pressure for AR. Therefore, studies demonstrating differences in resistance/resilience of ruminants to helminths can help develop appropriate strategies to control parasitism by using resistant breeds and improving sustainable rearing of local breeds (Zanzani *et al*., 2014). Goats can be significantly more heavily infected than sheep, and both acquisition and expression of immune responses against GINs are less efficient in goats than in sheep (Hoste *et al*., 2010). Therefore, studies evaluating this issue in goats are of particular interest. In a study by Alberti *et al*. (2014), Alpine goats (AB), a cosmopolite and high milk producing breed and Nera di Verzasca (NV), an Italian autochthonous goat breed, reared in a mountain ecosystem, were tested throughout 1 lactation season for the effect of natural GIN infections on yield and quality of their milk by FEC. While GIN infections resulted in a reduction in milk yield, protein and fat contents in both breeds, the effects were more pronounced in the AB compared with the autochthonous goat breed (Alberti *et al*., 2014).

To further investigate the mechanism underlying these breed-associated differences, a second study was performed in AB and NV goats from the same farm in controlled nematode challenge as well as in natural GIN infection under field conditions, exploring explanatory variables associated with FEC and differences in anti-*T. circumcincta* antibody and packed cell volume (PCV) levels (Zanzani *et al*., 2020). In goats with experimental and natural GIN infection, mean EPG values were consistently lower in NV goats. Further, PCV was also influenced by the breed being higher in NV than AB goats. Lower PCV values were also associated with higher strongyle EPG in AB goats and the PCV decrease during GIN infection was likely due to the presence of *H. contortus*. Anti-*T. circumcincta* IgA levels were influenced by both strongyle EPG and breed, with IgA levels negatively associated with EPG. NV goats gave evidence of a more effective response to nematodes than the other breed, but the higher resistance to GIN infection seen in the NV breed did not appear to be related to circulating levels of *T. circumcincta*-specific IgA or IgE against L3. Therefore, both experimental and natural infection confirmed that NV goats are more resistant to infection with GINs. Another study (Agradi *et al*., 2022) demonstrated that reference haematological intervals in female goats of the local breed (NV) differed from those of the cosmopolitan breed (AB) under the same rearing conditions, with most red blood cell parameters being higher in NV than in AB, and that these differences indicated greater resistance to gastrointestinal parasites and adaptation to environmental stressors in the local breed, probably as a result of adaptation strategies to the breeding context developed over centuries.

A study by Zanzani *et al*. (2019) evaluated the quali-quantitative variations of FEC due to reinfection after an anthelmintic treatment in a naturally infected flock composed of 3 dairy goat breeds, an autochthonous (Orobic) and 2 cosmopolite (Alpine and Saanen) breeds, to investigate the influence of selected risk factors (sampling, breed, and number of births) on FEC, and to find the differences in GIN reinfection between autochthonous and cosmopolite goats. In cosmopolite goats, FEC was characterized by higher values in pluriparous than in primiparous animals, while an opposite trend of reinfection was observed in Orobic goats, possibly related to acquired immunity being stronger in the autochthonous than the cosmopolite breeds.

Selective breeding of sheep with increased resistance to GINs has been suggested for sustainable control of parasite infections, and genetic variation between individuals and breeds has been documented in many sheep breeds (Bishop and Morris, 2007). The Sarda sheep breed is the most important Italian dairy sheep breed with about 3 million animals in about 10 000 flocks (Casu *et al*., 2022). Farming systems vary from semi-extensive to semi-intensive with a widespread use of grazing on natural pastures and forage crops where infection by GINs is unavoidable. A high positive correlation was found between FEC under artificial or natural infection (Aguerre *et al*., 2018; Gruner *et al*., 2004). This evidence and the estimate of heritability found in an Italian study (Casu *et al*., 2022) in a large experimental population suggest that genetic selection for parasite resistance could be considered in the Sarda breed and provided a list of candidate genes and polymorphisms that could be used in further validation studies.

Nematode parasites and mastitis are the major animal health constraints in dairy sheep with a major impact on productivity. Somatic cell count (SCC) has been used as an indicator to select for increased resistance to subclinical mastitis (Barillet, 2007). Implementation of nematode resistance and SCC in breeding programs requires the knowledge of the relationships between these major health traits. The genetic (co)variances of nematode parasites resistance (measured by FEC) under natural conditions of infection and SCC was estimated in dairy sheep in Italy (Sechi *et al*., 2009). In an experimental population consisting of 949 backcrossed ewes and 806 of their daughters, SCS and FEC records for each subpopulation were processed independently to compensate for specific environmental influences and to obtain lactation records for both traits that were used in the genetic analysis. The heritability of SCS in lactation (LSCS, arithmetic mean of test days per lactation) and FEC was 0.19 and 0.16, respectively. The genetic correlation was 0.21, whereas phenotypic correlation was 0.01. The estimated heritability confirmed that both traits could be selected using the classical quantitative approach. The estimated genetic correlation between LSCS and FEC indicated that selection for either trait would have no adverse effect on the other.

Therefore, the implementation of breeding strategies aimed at obtaining animals with an inherently low susceptibility to nematode infections could play an increasingly important role in the integrated control of these helminths in small ruminants. In Italy, greater resilience to GIN has been demonstrated in local goat breeds, while in dairy sheep the possibility of selection for increased resistance to subclinical mastitis and GINs has been evaluated, and a list of candidate genes and polymorphisms has been presented for the Sarda breed that could be used in further studies.

## Conclusions and future directions

The numerous and different experiences carried out in Italy to develop sustainable management of gastrointestinal helminths in small ruminants have been described for the first time in this review, with the aim of identifying best practices and general recommendations. The peculiarities of the Italian small ruminant production system call for a tailored approach, which can be further differentiated according to the geographical location, the animal species and the type of production (meat or milk).

With the strategies of TST, the use of anthelmintics in ruminants can generally be reduced in a way that slows down the development of AR and maintains animal performance. However, there is still the need for more specific guidelines and thresholds regarding the indicators for TST. In the search for the optimal indicators, the number of studies conducted to date in Italian sheep and goat farms can be expanded to provide sound evidence of their effectiveness and feasibility. It is important to keep in mind that efficient indicators are not readily applicable in some contexts. For example, the use of production indicators may be difficult to introduce in farms characterized by low levels of management, so the use of BCS may be more appropriate. In addition, further trials should be conducted to develop a better understanding of the proportion of untreated animals required to maintain effective *refugia* under conditions of different parasite species, environments and animal husbandry systems, as previously observed by Kenyon *et al*. (2009). The appropriate knowledge of the epidemiology of the different parasites and of the distribution of AR strains throughout Italy is therefore an important preliminary step in the parasite control strategy.

Despite TST has been proven to reduce AR development, this approach is only used by a minority of small ruminant farmers, as shown by a recent survey among sheep farmers in UK who reported TST of ewes against GINs was performed on 29% of farms (Williams *et al*., 2021). As has already been done in the UK, a comprehensive understanding of farmer attitudes towards sustainable parasite control in Italy should be achieved in order to widely translate these principles into practice (Kenyon *et al*., 2009). Several initiatives are currently in play to enhance this process, e.g. TST approaches included in veterinary parasitology curricula; education programmes with agricultural stakeholder organization (Charlier *et al*., 2014) but certainly a major mentality shift would be required. Indeed, while all efforts should be addressed towards promoting TST, whole-flock treatments are likely to remain the main control strategy for the next future. This is currently considered acceptable in areas where AR is rare, such as in Italy, but farmers using TT should immediately switch to a TST approach when changes in the susceptibility of worm populations are detected (Cringoli *et al*., 2009; Charlier *et al*., 2014).

Finally, this review highlights the need for an overall strategy to change the attitude of Italian sheep and goat farmers and veterinary practitioners, towards a holistic approach to helminth control, integrating a careful use of chemical drugs with the adoption of new phytotherapeutic compounds and other alternative and integrative practices. The evidence and preliminary findings gathered and presented in this review may represent a first step towards a more structured national strategy to control helminth parasites in small ruminants.

## Data Availability

All reported data are available in this review.
